# Evidence of macrophage modulation in the mouse pubic symphysis remodeling during the end of first pregnancy and postpartum

**DOI:** 10.1038/s41598-020-68676-x

**Published:** 2020-07-24

**Authors:** B. G. Castelucci, A. H. M. Pereira, M. Fioramonte, M. F. Carazzolle, P. S. L. de Oliveira, K. G. Franchini, J. Kobarg, D. Martins-de-Souza, P. P. Joazeiro, S. R. Consonni

**Affiliations:** 1 Laboratory of Cytochemistry and Immunocytochemistry, Department of Biochemistry and Tissue Biology, Institute of Biology, State University of Campinas (UNICAMP), Campinas, Brazil; 2 Brazilian Biosciences National Laboratory, Brazilian Center for Research in Energy and Materials (CNPEM), Campinas, Brazil; 3 Laboratory of Neuroproteomics, Department of Biochemistry and Tissue Biology, Institute of Biology, State University of Campinas (UNICAMP), Campinas, Brazil; 4 Department of Genetics, Evolution, Microbiology and Immunology, Institute of Biology, State University of Campinas (UNICAMP), Campinas, Brazil; 5 School of Pharmaceutical Sciences, State University of Campinas (UNICAMP), Campinas, Brazil; 6 Experimental Medicine Research Cluster (EMRC), State University of Campinas (UNICAMP), Campinas, Brazil; 7 D’Or Institute for Research and Education (IDOR), São Paulo, Brazil

**Keywords:** Cell biology, Immunology, Proteomics, Transcriptomics, Reproductive biology

## Abstract

In mouse pregnancy, pubic symphysis (PS) remodels into an elastic interpubic ligament (IpL) in a temporally regulated process to provide safe delivery. It restores at postpartum to assure reproductive tract homeostasis. Recently, macrophage localization in the IpL and dynamic changes in the expression of inflammatory mediators observed from the end of pregnancy (D18, D19) to early days postpartum (1dpp, 3dpp) highlighted the necessity of the identification of the key molecules involved in innate immune processes in PS remodeling. Therefore, this study uses morphological and high-sensitivity molecular techniques to identify both macrophage association with extracellular matrix (ECM) remodeling and the immunological processes involved in PS changes from D18 to 3dpp. Results showed macrophage association with active gelatinases and ECM components and 25 differentially expressed genes (DEGs) related to macrophage activities in interpubic tissues from D18 to 3dpp. Additionally, microarray and proteomic analysis showed a significant association of interpubic tissue DEGs with complement system activation and differentially expressed proteins (DEPs) with phagocytosis, highlighting the involvement of macrophage-related activities in mouse PS remodeling. Therefore, the findings suggest that PS ECM remodeling is associated with evidence of macrophage modulation that ensures both IpL relaxation and fast PS recovery postpartum for first labor.

## Introduction

In mice, drastic modifications occur in the lower reproductive tract during pregnancy^1–5^. The pubic symphysis (PS) widens in a temporally regulated process to enlarge the space available for lower reproductive tract softening, ripening and expanding, providing both safe delivery of the offspring and maintenance of reproductive tract homeostasis^6,7^. During mouse pregnancy, PS extracellular matrix (ECM) remodeling gives rise to an interpubic ligament (IpL) that separates pubic bones and contributes to an optimal accommodation of the lower female reproductive tract^6–11^. After the first labor, PS histoarchitecture restoration provides mechanical stability to the reproductive tract and the ability to initiate and maintain subsequent pregnancies^12–14^.

Aberrant modifications of PS ECM molecules directly alter tissue biomechanical behavior, leading to the development of pelvic organ prolapse^15^. Therefore, the remodeling of mouse PS through pregnancy and postpartum without loss of pelvic floor homeostasis requires a delicate balance between deposition and degradation of the ECM^7^. The increase in compliance and extendibility of the IpL before expected parturition on day 19 of pregnancy (D19) is due to the combined action of hormones and a wide variety of proteolytic enzymes, such as matrix metalloproteinases (Mmps), dipeptidyl peptidases (ADAMTs) and tissue inhibitor of metalloproteinases (Timps) and hyaluronidases (Hyals)^8,16^. These molecules change the levels of proteoglycan, hyaluronic acid (HA), collagen, elastic fibers and water of IpL, leading to the increase in compliance and extendibility of this ligament before parturition^8–10,17^. At IpL involution in postpartum, proteolytic enzymes and Hyals activity gradually decreased after 3dpp^8,16^, allowing bones and cartilage reformation by 10dpp to 40dpp at first pregnancy^12–14^.

Along with reproductive tissue remodeling, temporal changes in immune cell phenotypes reveal dynamic and multifaceted functions that are crucial to proper pregnancy development and successful labor^18–22^. Among immune cells, macrophages are involved in a wide range of gestational processes, including regulation of immune cell activities, placental cell invasion, initiation of labor and postpartum reproductive organ remodeling^19,23–29^. These cells show significant heterogeneity in function, majorly affected by their microenvironment. Under physiological conditions such as pregnancy connective tissue remodeling^30^, enzymatic breakdown of ECM components (proteoglycans and HA) produces soluble fragments that can act as damage-associated molecular patterns (DAMP). These endogenous signals trigger a fast macrophage response and interact with toll-like receptors (TLRs), leading to a sterile inflammation process to solve the adverse condition that initially led to DAMP release^31–33^. Interestingly, aberrant modifications in the mouse reproductive tract or pelvic floor EMC molecules seem to be intimately associated with increased pro-inflammatory cytokine production in pelvic organs^34^ and lead to exacerbated interpubic tissue relaxation for labor^15^.

In addition to recognizing the importance of immune cells in female reproductive tract modifications during pregnancy, studies showing immune cell involvement in PS remodeling in mice are scarce^12,14,20,35^. Although neither neutrophils nor eosinophils are associated with this process^12,20,35^, recent data showed that both pro-inflammatory (M1) and anti-inflammatory (M2) activated macrophage phenotypes are present in the IpL from labor to early postpartum, suggesting that the differential gene expression of inflammatory mediators found in mouse interpubic tissues during this period may reflect IpL macrophage activities^36^. Although significant PS ECM turnover gives rise to ECM fragments, it is still unknown whether macrophages interact with extracellular DAMPs or actively participate in IpL ECM remodeling for labor and postpartum recovery.

The absence of mouse PS widening in the late stage of pregnancy leading to abnormal parturition and dystocia was observed in both genetically manipulated relaxin and relaxin receptor genes^37–39^. Interestingly, a similar female mouse phenotype was also reported under combined ablations of macrophage mannose (ManR, *Mrc1*) and asialoglycoprotein (AsgR, *Asgr1/2*) receptors^40^. These scavenger-associated receptors confer biological functions mainly associated with cell migration and adhesion to monocytes and macrophages^41^. Though those results suggest that depletion of these macrophage-related receptors is related to PS dysfunction during labor, it is not clear which ones are the signaling molecules and which biological processes are related to immune response pathways from late pregnancy to postpartum.

Considering the role that innate immunity functions may play in this remodeling of the mouse PS, the aim of the present study is to identify both macrophage association with ECM remodeling and the immunological processes involved in PS changes during the end of first pregnancy and postpartum using morphological and high-sensitivity molecular techniques. Double immunostaining and in situ zymography results showed an association of cells positive for macrophage marker (F4/80) with active gelatinases from D18 to 3dpp and F4/80^+^ cells co-localization with immunomodulatory ECM components decorin, HA and versican in the IpL, mainly at 1dpp and 3dpp. In agreement with morphological data, microarray results identified 25 differentially expressed genes (DEGs) related to macrophage activities in interpubic tissue remodeling from D18 to 3dpp. Microarray and proteomic analyses also demonstrated a significant association of interpubic tissue DEGs with complement system activation and differentially expressed proteins (DEPs) with phagocytosis. Then, our study sheds light on the interplay of macrophage-related innate immune system processes in mice interpubic tissue remodeling from the end of pregnancy to postpartum.

## Results

### Dynamic remodeling of mouse PS at the end of pregnancy and early postpartum

**Figure 1:**
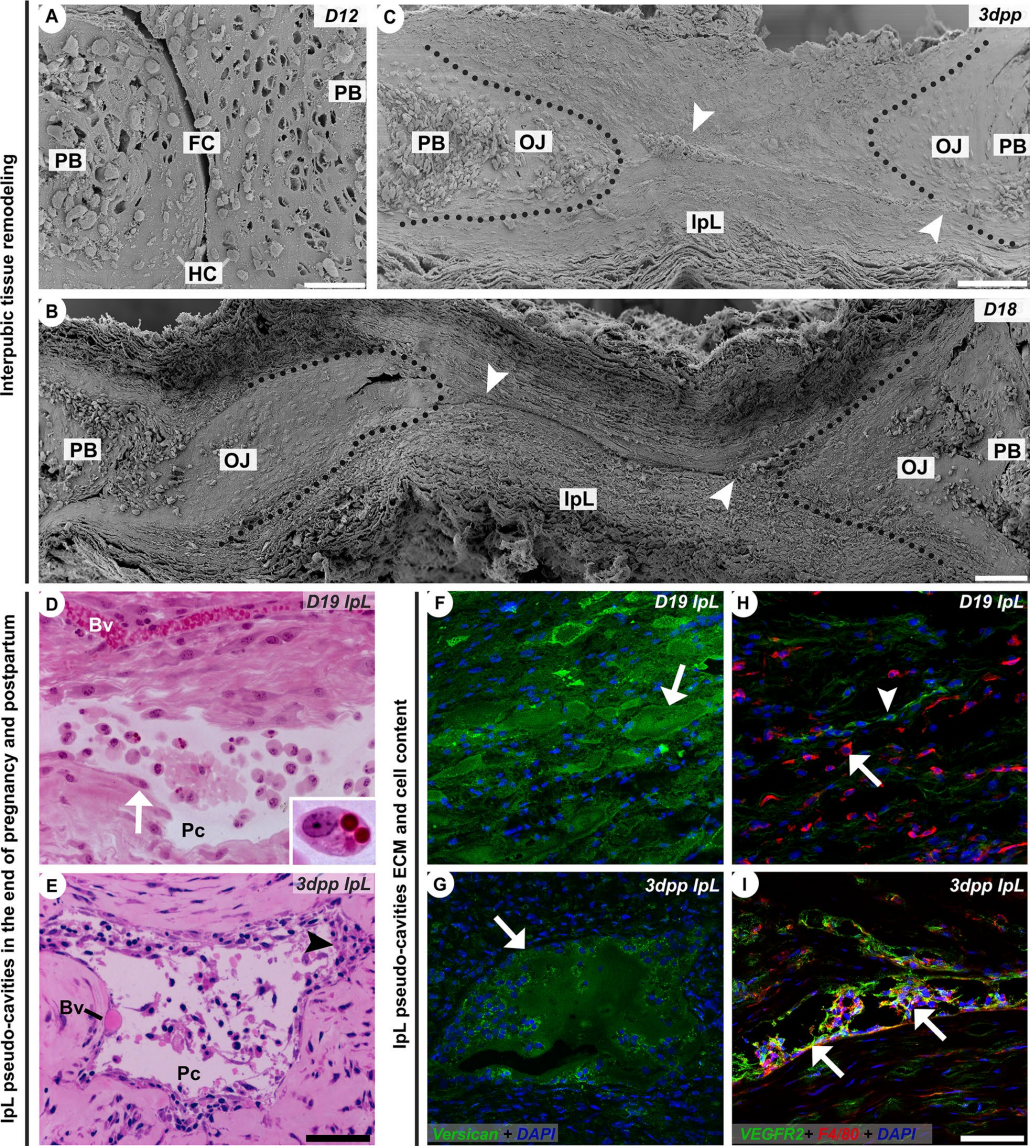
Morphological aspects, ECM, and cellular content of interpubic tissues and IpL pseudo-cavities during pregnancy and postpartum. (**A**–**C**) Scanning electron microscopy of PS and IpL transverse sections. (**A**) D12 PS fibrocartilaginous disc (FC) attached to hyaline cartilaginous pads (HC) continuous with pubic bones (PB). (**B**,**C**) D18 relaxed, and 3dpp remodeled IpL containing pseudo-cavities (white arrowhead) closed the PB and osteoligamentous junctions (OJ), delimited by a grey dot line. (**D**,**E**) Light micrographs of pseudo-cavities (Pc) in both D19 relaxed and 3dpp remodeled IpL haematoxylin-phloxine B stained sections, which differ strikingly from well-preserved blood vessels (Bv). Note that in well-preserved resin-embedded specimens, the predominance of recruited mononuclear phagocyte-like cells (arrowhead) that may engulf material in the cytoplasm (detail) inside of pseudo-cavities in both D19 relaxed and 3dpp remodeled IpL. (**D**) D19 relaxed IpL pseudo-cavity border lined with a discontinuous layer of fibroblast-like cells (arrow) directly attached to IpL fibrous ECM. (**E**) 3dpp remodeled IpL pseudo-cavity border with more than one layer or discontinuous cell clusters associated with a dense collagen matrix (arrowhead). (**F**–**I**) Confocal immunolocalization of versican, VEGFR2^+^ cells, and F4/80^+^ cells present in pseudo-cavities and IpL tissue, counterstained with DAPI (blue). (**F**,**G**) versican V0 variant core protein immunodetection on D19 relaxed IpL ECM and inside of 3dpp remodeled IpL pseudo-cavity (green; marked with arrows), where immunolocalization was most intense surrounding recruited mononuclear phagocyte-like cells. (**H**,**I**) Double staining of F4/80 with VEGFR2 showed that F4/80^+^-recruited phagocytes (red; marked with an arrow) do not co-localize with VEGFR2^+^ cells (green; marked with arrowhead) on D19 relaxed IpL, but F4/80^+^/ VEGFR2^+^ double-positive cells lining the 3dpp remodeled the IpL pseudo-cavity border (yellow; marked with arrows). (**A**) Scale bar = 50 μm. (**B**,**C**) Scale bars = 100 μm. (**D**,**E**) Scale bars = 30 μm. (**F**–**I**) Scale bars = 30 μm.

At the end of pregnancy and early postpartum, PS remodeling results in a relaxed IpL that has pseudo-cavities (Fig. 1A–C) filled with non-fibrillar versican/V0 variant-rich ECM (from now on referred as versican) and F4/80^+^ cells (Fig. 1F–I). After 3dpp, the IpL rapidly remodels to tie the pelvic bones together in a near non-pregnant fibrocartilaginous joint (Fig. 1C), as it was described in previous studies^6,14,42^.

Additionally, at both D19 and 3dpp, IpL pseudo-cavities had their borders lined mainly by mononuclear phagocyte-like cells positive for F4/80 macrophage markers (Fig. 1D,E,H,I). Those mononuclear phagocyte-like cells also presented phagocytic material in their cytoplasm, showing the phagocytic activity in the IpL (Fig. 1D). Double immunostaining assay showed that while only single F4/80^+^ cells were immunolocalized at D19 pseudo-cavity borders (Fig. 1H), double-positive F4/80^+^/VEGFR2^+^ cells were localized at 3dpp pseudo-cavities (Fig. 1I), indicating that two distinct F4/80^+^ cells populations might be involved in the pseudo-cavity organization at D19 and 3dpp.

### F4/80^+^ cells associated with active gelatinases and immunomodulatory ECM components during interpubic tissue remodeling

**Figure 2:**
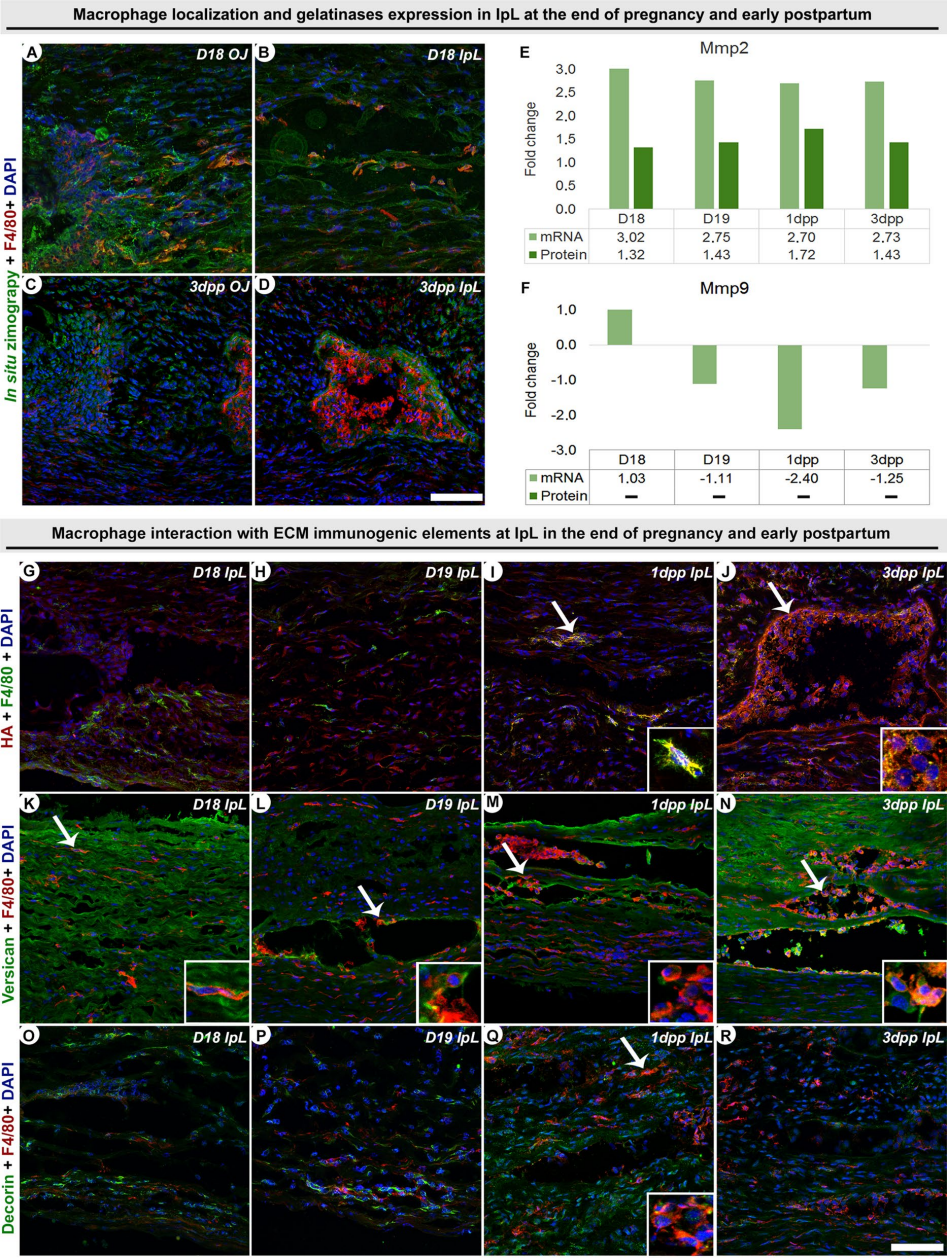
F4/80^+^ cells co-localize with gelatinase and ECM elements during interpubic tissue remodeling from D18 to 3dpp. (**A**–**D**) Positive fluorescent labeling of gelatinase activity (green) and F4/80^+^ cell immunolocalization (red) at osteoligamentous junction (OJ) and the interpubic ligament (IpL) at D18 (**A**,**B**) and 3dpp (**C**,**D**). F4/80^+^ cells co-localized with active gelatinase all over interpubic tissues at D18 and presented marked co-localization inside IpL pseudo-cavities at 3dpp. (**E**,**F**) Quantitative data of gene expression and protein production levels of metalloprotease (Mmp) with gelatinase activity (Mmp2-9) in interpubic tissues from D18 to 3dpp. While Mmp2 mRNA and protein levels were remarkably upregulated throughout interpubic tissue remodeling (**E**), the Mmp9 mRNA was downregulated, and its protein production was not detected from D18 to 3dpp (**F**). Data obtained by microarray and shotgun proteomic analysis, with a *p* ≤ 0.05 indicating statistical significance. (**G**–**R**) Double immunostaining for F4/80 macrophage marker and immunomodulatory ECM components at the interpubic ligament from D18-3dpp. (**G**–**J**) Hyaluronic acid (HA) co-localized with F4/80^+^ cells (details and arrows) in the periphery and edges of IpL pseudo-cavities at (**I**) 1dpp and (**J**) 3dpp. (**K**–**N**) Double-positive cells for both versican (V0 variant core protein) and F4/80 (details and arrows) were dispersed in the ECM, at the interior and edges of the pseudo-cavities and in the IpL from D18 to 3dpp. (**O**–**R**) Decorin co-localized only with F4/80^+^ cells (detail and arrow) at the periphery and edges of IpL pseudo-cavities at 1dpp. (**A**–**D**) Scale bars = 50 μm. (**G**–**R**) Scale bars = 30 μm.

Considering that enzymatic activity at interpubic tissue remodeling leads to ECM structural changes^8,16^ and that some ECM components can modulate macrophage differentiation and the function of Mmps^32^, we investigated the co-localization between F4/80^+^ cells and HA, versican, decorin or active gelatinases at the end of pregnancy and early postpartum. Additionally, to identify modifications in gelatinase production in interpubic tissue at this period, we analyzed Mmp-2 and -9 gene expression and protein production by microarray and proteomics (Fig. 2).

Confocal results showed that F4/80^+^ cells co-localize with gelatinases (Fig. 2A–D) and with a distinct set of ECM components during interpubic tissue remodeling (Fig. 2G–R) from D18 to 3dpp. Together with in situ zymography identification of F4/80^+^ cells and gelatinase association at the end of pregnancy and postpartum, Mmp2 mRNA and protein levels were remarkably upregulated throughout interpubic tissue remodeling (Fig. 2E,F). From D18 to D19, only versican co-localized with F4/80^+^ cells in IpL (Fig. 2G,H,K,L,O,P). At postpartum, F4/80^+^ cells co-localized with all ECM elements analyzed at 1dpp and with HA and versican at 3dpp (Fig. 2I,J,M,N,Q,R).

### Immune system biological processes involved in interpubic tissue remodeling

**Figure 3:**
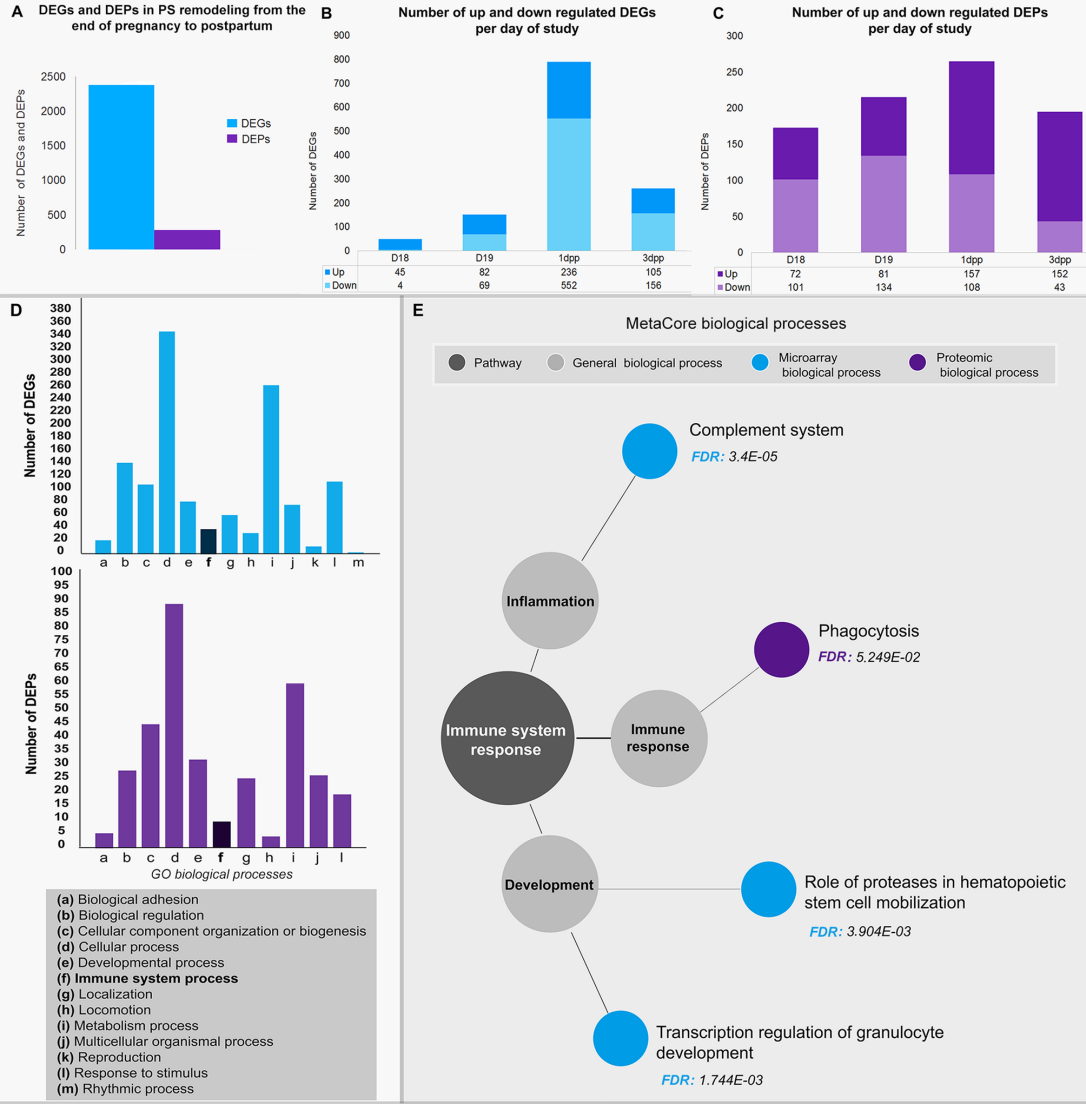
Global analysis of differentially expressed genes (DEGs) and proteins (DEPs) involved in interpubic tissue remodeling and enrichment analysis of microarray and proteomic data in immune system-related processes. (**A**) Genes and proteins were differentially regulated during interpubic tissue remodeling from D18 to 3dpp, with 2,379 DEGs and 280 DEPs involved in this process. (**B**,**C**) The total number of DEGs and DEPs progressively increased from D18 to 1dpp and decreased at 3dpp. Upregulated DEGs were abundant on D18, whereas positive DEPs were observed on 1dpp. Downregulated DEGs were abundant on 1dpp, while DEPs were more frequent at 3dpp. (**D**) PANTHER classification of biological processes by The Gene Ontology (GO) (a–m) associated with DEGs or DEPs identified by microarray and shotgun proteomic analysis during interpubic tissue remodeling. (**E**) MetaCore enrichment analysis for biological processes was completed for all time points and distributed the statistically significant data (*p* < 0.01/minimum FDR < 10^–2^) for at least one day of study into processes related to immune system. The statistically significant biological processes were grouped by the authors before being displayed in the diagram.

To evaluate how interpubic tissue DEGs and DEPs are involved in the immune system canonical pathways and biological processes during both IpL relaxation and postpartum remodeling from D18 to 3dpp, we utilized a relative analysis of transcriptomic and proteomic data. DEGs and DEPs (fold changes between 2 and − 2, *p* < 0.05) of whole interpubic tissues from D18 to 3dpp were obtained after normalization to D12 levels for use in enrichment data analysis (Fig. 3A).

Results from the global analysis indicated that DEGs and DEPs varied over distinct time points and 1dpp presented the highest amount of DEGs and DEPs (Fig. 3B,C). Enrichment analysis performed using the PANTHER classification system (https://www.pantherdb.org) correlated both DEGs and DEPs to the “immune system process” during interpubic tissue remodeling (Fig. 3D). Also, enrichment analysis by the MetaCore database showed that DEGs and DEPs were associated with distinct immune system response processes. However, while DEGs were significantly included in both the complement system inflammation process and development-related processes, DEPs were included only in the immune response of phagocytosis (Fig. 3E).

**Figure 4:**
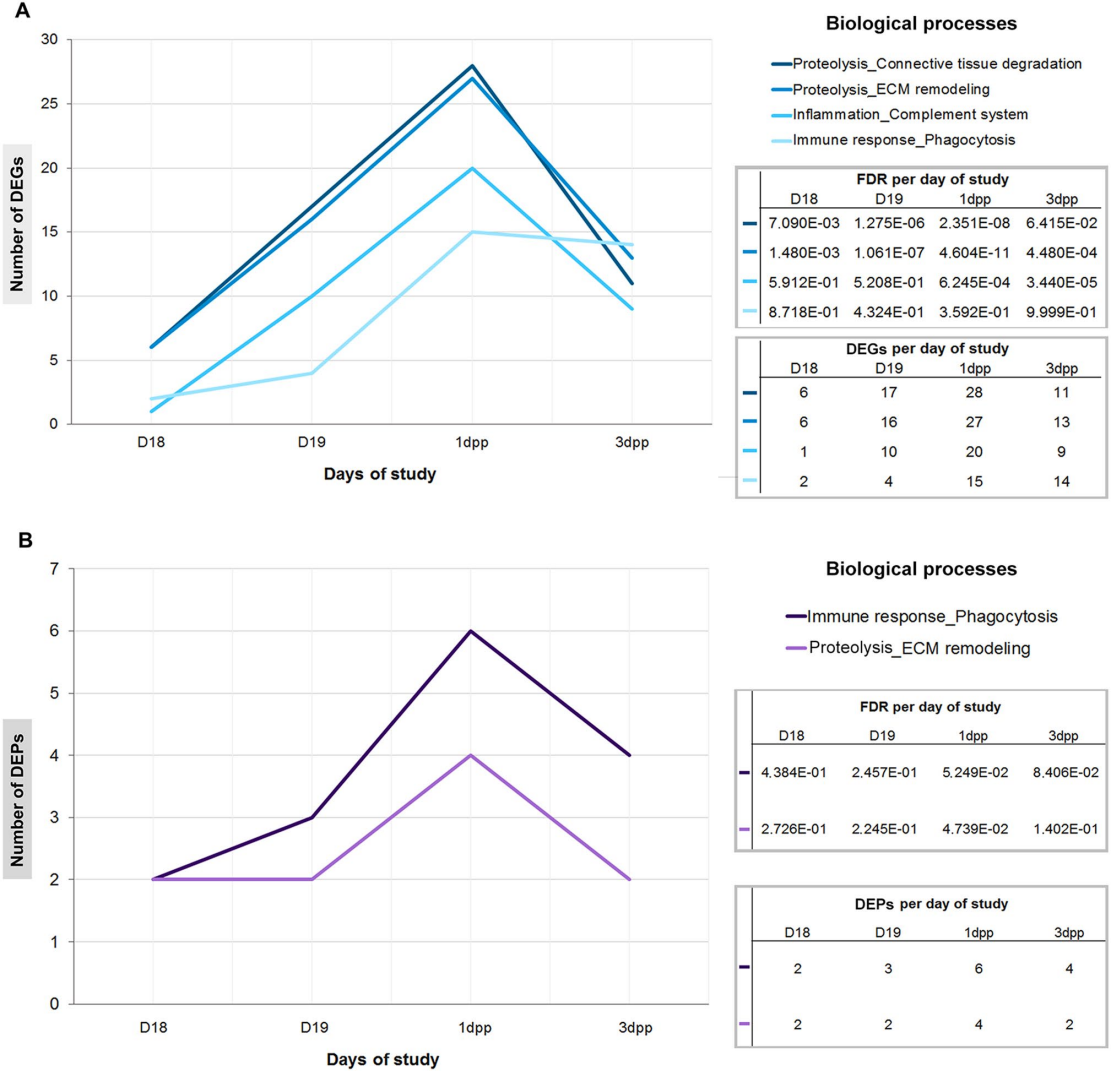
Enrichment analysis of microarray and proteomic data in biological processes by day of study. (**A**) Number of DEGs grouped from D18 to 3dpp. (**B**) Number of DEPs grouped from D18 to 3dpp. Note that 1dpp presented the highest amount of both DEPs and DEGs linked to immune system-related processes.

When analyzed per time point, the number of DEGs and DEPs associated with the immune system-related processes was higher at postpartum than at the end of pregnancy (Fig. 4A,B). The increased number of DEGs and DEPs associated with interpubic tissue remodeling at early postpartum was followed by expressive false discovery rate (FDR) and *p values* for each process listed by the MetaCore database (see Supplementary Table S1 online). Although the relationship between DEGs and DEPs at the listed immunological processes was indicated from D18 to D19 of pregnancy by the MetaCore database, these associations were not statistically significant (*p* > 0.05 and FDR > 10^–2^).

**Figure 5:**
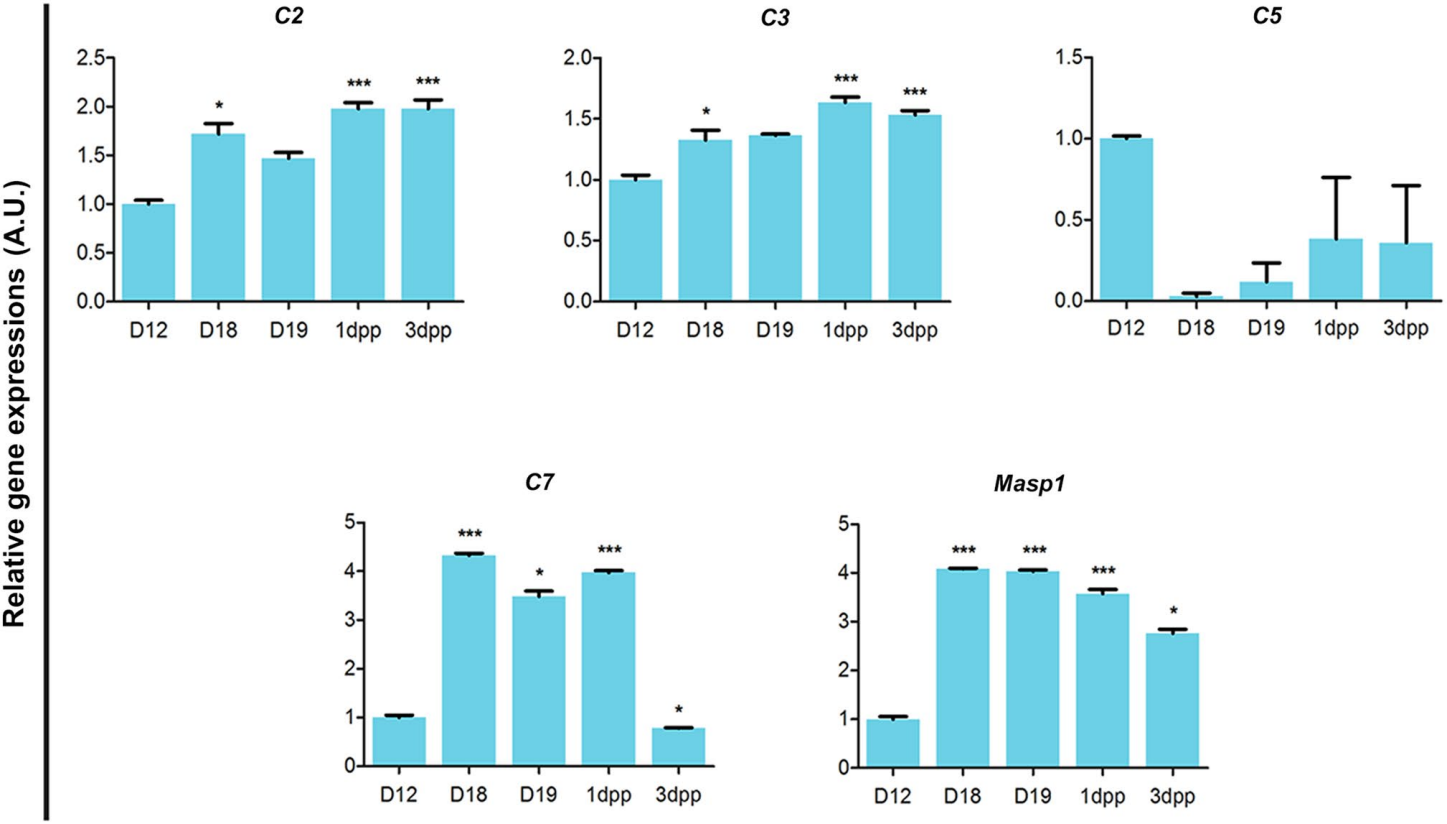
Dynamic changes in complement system-related DEG expression levels at interpubic tissue remodeling from D18 to 3dpp by qPCR. A.U. = arbitrary unit. One-way ANOVA with Tukey’s host test (*p < 0.05 vs. D12, ** *p* < 0.01 vs. D12, ***p < 0.0001 vs. D12). (D12 n = 6, D18 n = 3, D19 n = 3, 1dpp n = 3, 3dpp n = 6).

Concerning immune response-related processes, the "transcriptional regulation of granulocyte development" (see Supplementary Table S2 online) and the "role of proteases in hematopoietic stem cell mobilization" (see Supplementary Table S3 online) indicated most of DEGs were downregulated. However the "complement system" indicated most of DEGs were upregulated (Table 1). Expression levels from the microarray analysis for some of the complement system-related genes were confirmed by qPCR, suggesting that the increased number of complement system-related DEGs were involved in IpL remodeling from D18 to postpartum (Fig. 5).

**Table 1:** List of DEGs (− 2 > fold changes > 2) associated with the complement system by MetaCore enrichment analysis for biological processes, detailing their molecular function and fold changes from D18 to 3dpp (n = 3/group; FDR < 10^–3^; *p* < 0.0001).

Ensembl number	Gene ID	Gene name	Molecular function	Fold change			
				D18	D19	1dpp	3dpp
ENSMUST00000046384	*C1qb*	Complement component 1, q subcomponent, beta polypeptide	Generic protease	–	–	2.57	2.18
ENSMUST00000184647	*C1rb*	Complement component 1, r subcomponent B	Generic protease	–	–	2.25	2.40
ENSMUST00000159143	*C1s1*	Complement component 1, s subcomponent 1	Generic protease	–	–	3.12	3.31
ENSMUST00000025230	*C2*	Complement component 2 (within H-2S)	Generic protease	–	–	2.11	2.05
ENSMUST00000024988	*C3*	Complement component 3	Generic binding protein	–	–	2.39	2.12
ENSMUST00000042081	*C3ar1*	Complement component 3a receptor 1	GPCR	–	2.35	4.02	3.34
ENSMUST00000110689	*C7*	Complement component 7	Generic binding protein	6.04	4.29	5.69	–
ENSMUST00000061653	*Cfd*	Complement factor D (adipsin)	Generic protease	–	− 2.20	–	–
ENSMUST00000028179	*Fcnb*	Ficolin B	Generic binding protein	–	–	− 6.10	− 2.16
ENSMUST00000089883	*Masp1*	Mannan-binding lectin serine peptidase 1	Generic protease	4.92	5.63	3.68	2.27
ENSMUST00000131456	*Serping1*	Serine (or cysteine) peptidase inhibitor, clade G, member 1	Generic binding protein	–	–	2.48	2.49

Once complement system activation and phagocytosis were found to be directly related to macrophage roles in homeostatic tissue remodeling processes^43^, an analysis of function designations for mice was performed using the DAVID (https://david.abcc.ncifcrf.gov) database to identify interpubic tissue DEGs and DEPs directly related to macrophage activation, differentiation, function and regulation. The analysis identified 25 DEGs related to macrophage activities (Table 2) and none DEPs. Analyzing immune system-related processes, 13 DEPs were found, showing that although no directly related macrophage DEPs were observed in interpubic tissue remodeling, the modification in DEGs may reflect a signal for immune system-related protein production during this remodeling process (Table 3). In agreement with the hypothesis, our data showed that from D18 to 3dpp, DEGs directly related to macrophages were mainly grouped into processes, such as regulation of macrophage activation, macrophage activation, positive regulation of macrophage chemotaxis, positive regulation of macrophage differentiation and macrophage differentiation (Table 2). Similarly, DEPs were grouped into processes related to macrophage activity, such as immune system response to stimulus, antigen presentation and innate immune response differentiation (Table 3).

**Table 2:** List of DEGs (− 2 > fold changes > 2) directly related to macrophage biological processes or functions at DAVID database and their fold changes from D18 to 3dpp. (n = 3/group).

Ensembl number	Gene ID	Gene name	Biological process or function	Fold change			
				D18	D19	1dpp	3dpp
ENSMUST00000052172	*Cxcr4*	Chemokine (C-X-C motif) receptor 4	Cell migration, cellular response to cytokine stimulus	–	–	− 3.39	–
ENSMUST00000073043	*Cxcl12*	Chemokine (C-X-C motif) ligand 12	Chemotaxis, inflammatory response	–	–	− 2.56	–
ENSMUST00000028045	*Mrc1*	Mannose receptor, C type 1/Macrophage mannose receptor 1	Endocytosis	–	–	2.43	–
ENSMUSG00000035352	*Ccl12*	Chemokine (C–C motif) ligand 12	Macrophage chemotaxis	–	3.45	4.31	–
ENSMUST00000100487	*Eif2ak1*	Eukaryotic translation initiation factor 2 alpha kinase 1	Macrophage differentiation	–	–	− 2.13	− 2.10
ENSMUST00000035323	*Spib*	Spi-B transcription factor (Spi-1/PU.1 related)	Macrophage differentiation	–	–	− 2.77	− 2.63
ENSMUST00000015003	*E2f4*	E2F transcription factor 4	Macrophage differentiation, mitotic cell cycle	–	–	− 2.06	–
ENSMUST00000045288	*Tgfb2*	Transforming growth factor, beta 2	Negative regulation of macrophage cytokine production	2.09	2.05	2.68	–
ENSMUST00000003687	*Tgfb3*	Transforming growth factor, beta 3	Negative regulation of macrophage cytokine production	–	–		2.39
ENSMUST00000021028	*Itgb3*	Integrin beta 3	Negative regulation of macrophage derived foam cell differentiation	–	–	− 3.75	–
ENSMUST00000114268	*Snca*	Synuclein, alpha	Positive regulation of macrophage activation	− 4.02	− 4.08	− 6.76	− 6.48
ENSMUST00000020668	*Havcr2*	Hepatitis A virus cellular receptor 2	Positive regulation of macrophage activation	5.14	3.43	2.16	–
ENSMUST00000025724	*Il33*	Interleukin 33	Positive regulation of macrophage activation	–	–	2.11	–
ENSMUST00000016168	*Lbp*	Lipopolysaccharide binding protein	Positive regulation of macrophage activation	3.26	2.47	2.45	–
ENSMUST00000033004	*Il4ra*	Interleukin 4 receptor alpha	Positive regulation of macrophage activation	3.81	3.83	3.20	–
ENSMUST00000042081	*C3ar1*	Complement component 3a receptor 1	Positive regulation of macrophage chemotaxis	–	2.35	4.02	3.34
ENSMUST00000015460	*Slamf1*	Signaling lymphocytic activation molecule family member 1	Positive regulation of macrophage chemotaxis	–	–	− 2.35	–
ENSMUST00000000193	*Ccl2*	Chemokine (C–C motif) ligand 2	Positive regulation of macrophage chemotaxis	–	2.53	–	–
ENSMUST00000046186	*Spon2*	Spondin 2, extracellular matrix protein	Positive regulation of macrophage cytokine production	3.14	4.79	–	–
ENSMUST00000031320	*Pf4*	Platelet factor 4	Positive regulation of macrophage derived foam cell differentiation	–	–	− 2.36	− 2.30
ENSMUST00000022701	*Rb1*	Retinoblastoma 1	Positive regulation of macrophage differentiation	–	–	− 2.45	–
ENSMUST00000084032	*Adam9*	Adisintegrin and metallopeptidase domain 9 (meltrin gamma)	Positive regulation of macrophage fusion	2.22	2.12	2.02	–
ENSMUST00000024988	*C3*	Complement component 3	Positive regulation of phagocytosis, complement activation, positive regulation of apoptotic cell clearance	–	–	2.39	2.12
ENSMUST00000005950	*Mmp12*	Matrix metallopeptidase 12/Macrophage metalloelastase	Proteolysis	–	–	2.26	–
ENSMUSG00000032238	*Rora*	RAR-related orphan receptor alpha	Regulation of macrophage activation	–	2.28	2.10	–

**Table 3:** List of DEPs (− 2 > fold changes > 2) related to immune system biological processes at DAVID database and their fold changes from D18 to 3dpp. (n = 3/group).

Uniprot ID	Protein symbol	Protein name	Biological process or function	Fold change			
				D18	D19	1dpp	3dpp
P31725	Calgranulin B	S100 calcium binding protein A9 (calgranulin B)	Immune system process, response to stimulus, leukocyte migration involved in inflammatory response	–	–	− 2.71	–
Q60710	SAMHD1	Deoxynucleoside triphosphate triphosphohydrolase	Innate immune response, regulation of innate immune response, negative regulation of type I interferon-mediated signaling pathway	− 4.97	− 4.31	− 3.43	–
P04186	CFB	Complement factor B	Immune system process, response to stimulus, leukocyte migration involved in inflammatory response, complement activation, alternative pathway	–	− 2.36	− 2.49	− 3.41
E9PV24	FGA	Fibrinogen alpha chain	Immune system process, response to stimulus	–	2.25	2.04	–
P01899	H2-D1	H-2 class I histocompatibility antigen, D-B alpha chain	Antigen processing and presentation of endogenous peptide antigen via MHC class Ib	–	− 3.35		–
P03991	H2-K1	H-2 class I histocompatibility antigen, K-B alpha chain	Antigen processing and presentation of endogenous peptide antigen via MHC class Ib	–	− 3.35	–	–
P01898	H2-Q10	H-2 class I histocompatibility antigen, Q10 alpha chain	Antigen processing and presentation of endogenous peptide antigen via MHC class Ib	–	− 3.35	–	–
P14429	H2-Q7	H-2 class I histocompatibility antigen, Q7 alpha chain	Antigen processing and presentation of endogenous peptide antigen via MHC class Ib	–	− 3.35	–	–
P08071	LTF	Lactotransferrin	Immune system process, response to stimulus, positive regulation of toll-like receptor 4 signaling pathway	− 7.73	− 54.67	–	–
P11672	LCN2	Neutrophil gelatinase-associated lipocalin	Innate immune response, cellular response to tumor necrosis factor, cellular response to interleukin-1	− 2.15	− 2.76	–	–
Q5I2A0	SERPINA3G	Serine protease inhibitor A3G	Apoptotic process, response to cytokine, adaptive immune response	–	− 3.12	–	–
Q7TN37	TRPM4	Transient receptor potential cation channel subfamily M member 4	Homeostatic process, immune system process, response to stimulus, regulation of T cell cytokine production	–	–	2.63	2.10
P27548	CD40LG	CD40 ligand	Immune system process, response to stimulus	–	–	–	2.65

Although the number of macrophage-related DEGs (Table 2) gradually increased from the end of pregnancy (D18 = 7 DEGs; D19 = 11 DEGs) to the first day after parturition (1dpp = 22 DEGs) in a similar profile observed at global analysis, DEPs (Table 3) were more abundant and markedly downregulated at D19, decreasing in number at postpartum (1dpp = 5 DEPs; 3dpp = 3DEPs). We also noticed that processes with inverse functions, such as “positive regulation of macrophage cytokine production” and “negative regulation of macrophage cytokine production,” had exclusively upregulated DEGs. Additionally, "positive regulation of macrophage activation" processes presented both positive and negatively regulated macrophage-related DEGs from the end of pregnancy to postpartum recovery (Table 2), highlighting a constant balance of macrophage-related gene activities during interpubic tissue remodeling. Complement system elements were listed as directly related to both macrophage DEGs (Table 3—*C3* and *C3a*) and DEPs related to immune system processes such as phagocytosis (Table 2—CFB). This result suggests the possibility of macrophage and complement system interactions for IpL relaxation at the end of pregnancy and PS recovery postpartum.

## Discussion

This paper highlights that PS ECM remodeling is associated with the temporal activation of innate immune response processes that may lead to the modulation of macrophage activity. This association has been demonstrated in a homeostatic remodeling of interpubic tissues that leads to safe delivery and fast PS histoarchitecture recovery after first labor, granting reproductive tract and pelvic floor integrity.

Our light microscopy and SEM results illustrated the classical remodeling of interpubic tissues with pubic bone separation in labor by IpL relaxation^7^, as well as at the beginning of the postpartum restoration, which, in approximately 40 days, will give rise to a structure similar non-pregnant histoarchitecture^12^. Our morphological analysis also showed non-vascular pseudo-cavities in the IpL, filled with non-collagen ECM that presented similarities with previously described IpL sinusoidal like-cavities^14^ or symphyseal space^6^ with indistinct edges, composed mainly of newly differentiated (neutrophil 7/4^+^ cells) and mature macrophages (F4/80^+^)^36^. These IpL pseudo-cavities, which are easily distinguished from IpL ECM by their rich versican composition, are quite similar to the previous description of IpL sinusoidal like-cavities at the end of pregnancy^14^, which suggests that the plexus of large anastomosing cavities may accumulate fluids during the end of pregnancy, possibly helping IpL relaxation for labor^14^. Also, our results for versican V0 variant immunolocalization in IpL were in agreement with the high chondroitin and dermatan sulfated content in interpubic tissues from D18 to 3dpp and gene expression levels of *Vcan* (V0 and V1 variants) found from the end of pregnancy to postpartum^8^. The versican content and expression levels in interpubic tissues may have a direct relationship with the previously registered F4/80^+^ cell increase in IpL^36^, since activated macrophages can secrete V1 isoform of versican^44^. Interestingly, only at postpartum, when IpL pseudo-cavities seem to be a well-organized structure and not just a random network of cavities, our results of IpL F4/80^+^ cells co-localized with VEGFR2, showing that a heterogeneous macrophage population participates in interpubic tissue remodeling. These macrophages presented phenotypic similarity with cells that can form vascular mimicry networks in matrigel-like ECM^45^, indicating that IpL macrophages may be engaged in the pseudo-cavity organization during IpL ECM remodeling.

Although gene expression and proteomic assays agreed with previous findings of *Mmp2* expression in IpL from the end of pregnancy to postpartum, the decreased expression of *Mmp9* related to D12 shown by our microarray analysis pointed a distinct pattern from previous qPCR evaluations^16^. As in our proteomic analyses, previous blotting assessments of MMP9 production indicate low levels of MMP9 during PS remodeling^16^. These results highlighted that MMP2 is the main active form of gelatinase in the rich enzymatic *milieu* that conducts IpL remodeling. Consequently, it also determines that MMP2 was the active gelatinase detected in the in situ zymography experiments. Additionally, in situ zymography data showed that F4/80^+^ macrophages are one of the cells responsible for this active gelatinase production, first restrictedly associated with fibroblasts and chondrocytes^16^ during PS remodeling.

Our double immunofluorescent results showed that IpL F4/80^+^ cell co-localize mainly with versican at the end of pregnancy and with HA, versican and decorin at postpartum. Although the presence and content of these three ECM components are well characterized in mouse interpubic tissues during pregnancy and postpartum remodeling^8,11,17^, this is, to our knowledge, the first description of their association with F4/80^+^ cells. Although a small amount of pro-inflammatory low molecular weight (MW) HA had been previously registered in IpL at the end of pregnancy^8^, apparently no co-localization between F4/80^+^ cells and HA was observed in our confocal results from D18 to D19. In the same period, we registered F4/80^+^ cells and versican co-localization in IpL. Even though the increase in versican gene expression from D18 to D19 is not associated with increased levels of versican-degrading enzyme ADAMTS1, previous data point a constancy in chondroitin and dermatan sulfated content in the IpL at labor^8^. This event is parallel to high levels of both the Mmp2 gene and protein in the IpL from D18 to D19, indicating that MMP2 may similarly cleave IpL versican molecules as previous observations in rabbit lung^46^. Therefore, F4/80^+^ cells may co-localize with cleaved versican molecules at IpL at the end of pregnancy and labor. At postpartum, the co-localization between decorin, versican or HA with F4/80^+^ cells was simultaneous with the highest MMP2 protein levels detailed here and the formerly reported constant gene expression of *Hyal1* and *Hyal2* in IpL^8^, suggesting that these ECM elements may also be cleaved at postpartum.

MMP2 activity may release decorin and versican from the ECM to act as endogenous ligands of TLRs at macrophage, triggering sterile inflammation by enhancing the synthesis of the pro-inflammatory cytokines^31–33^. Interestingly, from D18 to D19, when F4/80^+^ cells co-localized with versican, both the presence of pro-inflammatory macrophages (F4/80^+^/CD40^+^)^36^ and high levels of NO, a marker of M1 activity^47^, were found in the IpL^48^. Additionally, from 1 to 3dpp, the co-localization of F4/80^+^ cells with decorin and versican is parallel to the previous register of increased levels of pro-inflammatory mediators in interpubic tissues^36^ and the upregulation DEGs related to macrophage activation in our microarray analysis. Keeping in mind that in its soluble form, digested ECM becomes potent DAMPs acting as immunomodulators^32,33^, our results strongly suggest that decorin and versican interact with IpL macrophage and influence their pro-inflammatory activation during fast IpL ECM degradation and PS histoarchitecture. Furthermore, macrophage co-localization with versican can also indicate that these cells produce versican in IpL, a process associated with macrophage maturation and activation in vitro^44,49^. Although both possibilities may occur in the IpL, the functional enrichment of microarray data reinforces the first hypothesis, as several DEGs related to the macrophage activation process were identified in interpubic tissues from D18 to D19, and negligible levels of DEGs directly associated with the macrophage differentiation process were also observed.

Unlike decorin and versican pro-inflammatory signals, HA interaction with macrophage can lead to effects in pro-inflammatory or anti-inflammatory stimuli according to MW^32^. During labor and early postpartum, IpL content is mainly composed of high MW products^8^, capable of inhibiting leukocyte production of pro-inflammatory mediators^31,50^. Coincidently, immunolocalization of both F4/80^+^ cells with HA at postpartum is concomitant with both the presence of M2 macrophages (F4/80^+^/TfR^+^) and high levels of interleukin 10 (*Il10*) gene expression registered in interpubic tissues^36^. These observations suggest that, on the one hand, degraded decorin and versican association with macrophages can result in M1 activation; on the other hand, IpL rich in high MW HA ECM may help in macrophage activity regulation by inducing an anti-inflammatory response. The presence of pro-inflammatory and anti-inflammatory signals provided by cleaved ECM components might explain the identification of upregulated DEPs related to the immune response to stimuli as well as both up- and down-regulated DEGs directly related to macrophage functional activities in IpL during postpartum. These expression patterns agree with the statement that the balance between pro- and anti-inflammatory signals along IpL remodeling regulates macrophage activity and ensures proper PS recovery at postpartum and the homeostasis of pelvic floor^36^.

As suggested by integrated data from gene and protein expression studies of cervical modifications at pregnancy and postpartum^51,52^, these similar essays may also help us to understand the immunological processes involved in mouse PS complex ECM remodeling at first pregnancy. Previous data^36,42^, along with our morphological analysis, support that immune system processes are statistically significant along with IpL remodeling by the enrichment analysis of microarray and proteomic experiments. Coincident with the highest number of F4/80^+^ cells and *F4/80* expression levels in IpL^36^, the highest number of DEGs and DEPs related to immune system-related processes found here during postpartum remodeling, particularly at 1dpp, also corroborate our F4/80^+^ cell and ECM component co-localization data. Macrophages may be essential for proper remodeling of interpubic tissues because *Mrc1* was found among our upregulated macrophage-related DEGs data in the IpL at 1dpp, while previous studies show the ablation of both *Mrc1* and *Asgr1/2* resulted in *IpL* relaxation failure, the arrest of labor and 61% of death in pregnant mice at parturition^40^.

The transcriptional regulation of granulocyte developmental processes from D18 to 3dpp in IpL shows constant inhibition of transcription factors (*Myb*, *Mxd1,* and *Gata1*), cell differentiation markers (*Csf3r* and *Ncf1*) and neutrophil activation markers (*Ltf, Elane, Prtn3, Mpo,* and *Cybb*), similarly with induced myeloid leukemia^53^ or genetically modified animal neutropenia^54^. Moreover, downregulation of DEGs (*Itga4, Cstg, Cxcl12,* and *Cxcr4*) in the role of proteases in hematopoietic stem cell mobilization processes is strongly associated with impaired transmigration, activation and survival of granulocytes^55,56^, and migration of other leukocytes in association with glycosaminoglycans^57^. These inhibitions are congruent with previous morphological descriptions that neither neutrophils nor eosinophils are recruited to remodel interpubic mouse tissues from pregnancy to postpartum^12,35^. Then, among immune system-related processes associated with PS remodeling, complement system activation and phagocytosis presented the highest FDR by MetaCore, and some DEGs, such as *C3* and *C3ar*, were directly associated with macrophage functions by DAVID analysis. The identification of phagocytosis as a significant process involved in IpL remodeling was also noted in light microscopy analysis and by recent electron microscopy data that showed phagocytic macrophage-like cells with phagosomes filled with engulfed materials in their cytoplasm, immersed in IpL ECM mostly at postpartum^36^.

Even though pregnancy complications, such as recurrent miscarriages, preterm birth and preeclampsia have been associated with excessive complement activation in animal models and humans at systemic levels^58^, local production of complement system components has indicated that complement might mediate distinct finely tuned roles in the context of tissue homeostasis and immunosurveillance in a context-driven fashion, independent of its systemic activities^43,59^. Except for *C7* and *serping1*, upregulated DEGs involved in complement system processes detected in IpL were previously shown to be expressed by naïve and different activated subsets of bone marrow macrophages, mainly by M0, M1 and M2b subsets, in vitro studies^60^. From D18 to 3dpp, cartilage and bone tissues undergo extended reabsorption and phenotypic modulation processes, leading to less-differentiated cell composition of osteoligamentous junctions from late pregnancy to early postpartum recovery^13^. This result decreases the possibility of previously reported production of *C1s*, *C2*, *C3ar* and *C3* by mature cartilage and bone cells^61^. The absence of *C5* and *C5ar* from D18 to 3dpp and the high levels of *C1q* in postpartum the IpL particularly strengthen the possibility of the local production of complement components by IpL macrophages, once naïve, or activated macrophages do not produce C5 in vitro, and bone marrow macrophages express higher levels of *C1q*^60^. According to this hypothesis, the increased local expression of complement system elements found in the IpL and the parallel increase in macrophage number and expression of macrophage-related cytokines^36^ suggest the existence of bidirectional feedback loops between complement elements and macrophage activation for proper PS remodeling.

Besides, although small osteoclasts were initially associated with pubic bone resorption at D14^14^, our results demonstrated that the *Ncf1* osteoclast maturation-related gene is downregulated in the IpL from D18 to 3dpp. Parallel to *Ncf1* downregulation, a progressive increase in *F4/80* expression in the IpL was previously described during this period^36^. Once NCF1 is an essential subunit of the ROS-producing NOX 2 complex that contributes to osteoclast formation by possibly regulating pre-osteoclast maturation^62^ and F4/80 acts as a suppressor of osteoclast maturation^63^, our results suggest that osteoclasts may not contribute to complement system component expression in interpubic tissues from D18 to 3dpp.

Keeping in mind that the complement system is critical to tolerogenic perception of apoptosis in many physiological processes^59,64^, the increase in both complement system activation DEGs and phagocytosis-involved DEPs shown by our enrichment analysis during the IpL remodeling process may be related to the regulation of apoptotic cell clearance. This is associated with the increase in cell death in interpubic tissues from labor to 5dpp^65^, phases of drastic remodeling of IpL and osteoligamentous junction components due to the breakdown of ECM to proper IpL relaxation for labor and fast restoration of PS histoarchitecture after delivery^7,13^. Similarly to complement system processes that can contribute to homeostasis by promoting tissue repair^43^, we found upregulation of initial elements of classical (*C1q, Cr1, Cs1, C2, C3, C3ar*) and lectin (*Masp1*) complement system pathways without the involvement of the C5-C5aR axis at IpL postpartum remodeling. These results are associated with early descriptions of increased *Il10* expression from 1 to 3dpp^36^ and are consistent with the immunological silent phagocytosis process mediated by C1q. In this process, the clearance of C1q and C3b-C3bi opsonized dying cells induces an anti-inflammatory program, increases the expression of immune checkpoint molecules and prevents the upregulation of maturation markers^64^. These results strengthen the hypothesis that complement system activation leads to phagocytic clearance of dying cells in PS remodeling.

As discussed above, PS remodeling involves drastic ECM modifications, including ECM component solubilization by enzymatic activities^7^. Interestingly, it was found that ECM components, such as soluble decorin and high MW HA^66,67^, and Mmps, such as MMP2 and MMP12^68,69^, can directly interact with complement components, impairing their activity and limiting tissue damage after injury. Coincidently, previous studies showed that the IpL is rich in decorin and high MW HA^8^ to the same period that increased *Mmp12* expression levels (1dpp), elevated amounts of MMP2 (D18 to 3dpp) and increased expression of complement system components were observed in this study, indicating that DEPs are only involved in phagocytosis processes. Considering that decorin, HA and MMP2 can decrease C1q activity^66–68^ and that MMP12 can degrade C3a^69^, it is possible that IpL ECM remodeling gives rise to a microenvironment that regulates both complement system activation and macrophage activity, assuring interpubic tissue remodeling without loss of homeostasis.

In summary, morphological and molecular findings suggest that mouse interpubic tissue modification for labor and postpartum recovery resembles the closure of a wound through an extensive tissue remodeling^59^, in which rigorous tissue remodeling involving protease-mediated degradation of ECM and rearrangements with intense cell turnover. This process induces the local release of danger signals that can activate immune cells, such as macrophages, into distinct phenotypes and modulate their function temporally from D18 to 3dpp. Active IpL macrophages release other immunomodulatory factors, such as cytokines and complement system elements, and activate innate immune pathways that can regulate the clearance of tissue turnover products (e.g., cell debris, apoptotic cells) without immune response activation, granting IpL relaxation for labor and fast mouse PS recovery to maintain pelvic floor integrity.

## Materials and methods

### Animals

Virgin female C57BL/6/JUnib mice (3 months old) were obtained from the Multidisciplinary Center for Biological Investigation on Laboratory Animal Science at UNICAMP. Mating and vaginal plug observation at day one of pregnancy (D1) were performed as described by Castelucci and coworkers^13^. Interpubic tissues obtained on days 12, 18, and 19 of pregnancy (D12, D18, D19), one and three days postpartum (1dpp, 3dpp) were arbitrarily distributed for morphological and molecular analyses. Three animals per group were used to obtain the interpubic tissues for light, electron and confocal microscopy analyses, and three (D18, D19, and 1dpp) or six (D12 and 3dpp) animals were used in the microarray, proteomic shotgun analysis, and real-time PCR analysis. In total, 102 animals were used for the experimental analyses. The animal experiments were conducted following the Guide for the Care and Use of Laboratory Animals issued by the National Institutes of Health (Bethesda, MD). All protocols using mice were approved by the Institutional Committee for Ethics in Animal Research (IB/UNICAMP, protocol 3789-1).

### Histology

PS and IpL samples were removed, fixed, and embedded in Historesin (Leica Microsystems, Heidelberg, Germany) as described by Consonni and coworkers^12^. The 3 μm width sections were stained with haematoxylin-phloxine B, according to Bennett and coworkers^70^, and then examined and imaged using a Nikon Eclipse E800 Microscope (Nikon Corporation, Tokyo, Japan).

### Scanning electron microscopy

Interpubic tissues from three animals (D12-3dpp) were fixed in 2.5% glutaraldehyde in a cacodylate 0.1 M solution with 4% paraformaldehyde (pH 7.4) and processed according to Joazeiro and coworkers^71^. Observations on the transverse plane through interpubic tissues were made under a JEOL Scanning Electron Microscope (JEOL, JEM 5800LV; Electron Microscopy Laboratory, IB/UNICAMP) at an accelerating voltage of 10 kV.

### Single and double immunostaining

For single immunostaining of versican (V0 variant core protein) and double immunostaining of F4/80 and VEGFR2, decorin or HA, interpubic tissues from three animals per day of study were frozen in n-hexane with liquid nitrogen and sectioned in the anteroposterior direction. The sections were fixed with acetone at − 20 °C for 3 min. Specifically for F4/80 and versican (V0 variant core protein) double immunostaining, samples were fixed in Carnoy's mixture (i.e., ethanol:chloroform:acetic acid 60:30:10 by volume) for 24 h at 4 °C and cryoprotected in a crescent sucrose gradient (10–30%) at 4 °C for 2 days. After the tissues were embedded in Tissue-Tek O.C.T. Compound (Sakura Finetek, USA, Inc.), they were finally frozen in n-hexane with liquid nitrogen and sectioned. Single versican immunostaining was performed according to Consonni and coworkers^9^, applying rabbit anti-mouse versican primary antibody (1:300/sc25831, Santa Cruz Biotechnology Inc., California, USA) and goat anti-rabbit secondary antibody (1:800/ab150077, AlexaFluor 488, Abcam plc., Cambridge, MA, USA). Double immunostaining was performed as described by Castelucci and coworkers^36^ using the antibodies described at Supplementary Tables S4 and S5 online. For both single and double immunostaining assays, nuclei were stained with DAPI for 5 min (SC-3598, Santa Cruz Biotechnology). Sections were mounted with VECTASHIELD Mounting Medium (Vector Labs, Burlingame, CA, USA), and samples were visualized with a Leica TCS SP8 (at Brazilian Biosciences National Laboratory, CNPEM) or Zeiss LSM 780-NLO (at National Institute of Science and Technology on Photonics Applied to Cell Biology-INFABIC, UNICAMP) confocal microscope using the 40 × oil objective.

### F4/80 immunostaining performed with in situ zymography

For the identification of F4/80^+^ cells associations with active gelatinases, F4/80 single immunostaining was associated with an in situ zymography assay. Reactions were performed in transverse cryosections in the anteroposterior direction (8 μm) of interpubic tissues frozen in n-hexane with liquid nitrogen. After fixation with acetone at − 20 °C for 3 min, in situ gelatin zymography was conducted with fluorescein-labeled DQ-gelatine from EnzChek Gelatinase/Collagenase Assay Kit (EnzChek, Molecular Probes, Eugene CA, USA) according to manufacturer’s recommendations and Bruni-Cardoso and coworker^72^. After gelatinase labeling, samples were incubated at room temperature with F4/80 primary antibody (1:300/T-2028.0100, BACHEM Biosciences, Inc., King of Prussia, PA) for one hour and subsequently incubated for 40 min with secondary antibody (1:500/ab150155, AlexaFluor 657, Abcam). After washing in 0.1 M PBS (pH 7.4) at 37 °C, nuclei were stained with DAPI for 5 min (SC-3598, Santa Cruz Biotechnology). Negative controls were incubated with DQ-gelatine in a substrate containing a 1% protease inhibitor cocktail (Sigma-Aldrich Company Ltd., UK) and without primary antibody. The sections were mounted with VECTASHIELD Mounting Medium (Vector Labs) and visualized with Zeiss LSM 780-NLO (National Institute of Science and Technology on Photonics Applied to Cell Biology-INFABIC, UNICAMP) using 40 × oil objectives.

### Microarray analysis

PS and IpL samples were removed and rapidly frozen in liquid nitrogen. Total RNA was extracted from D12 to 3dpp interpubic tissues following the RNeasy Mini Kit (QIAGEN, Hilden, Germany) manufacturer's instructions, and the RNA concentration was evaluated using a NanoDrop spectrophotometer, with the RNA concentration adjusted to approximately 1 μg/μl. Gene expression analysis was performed using the GeneChip Mouse Transcriptome Assay 1.0 Kit (Affymetrix, Thermo Fisher Scientific Inc., Massachusetts, USA). Microarray data analysis was performed using the R programming language. The linear model for microarray data analysis (LIMMA) was used to identify DEGs between D12 and other days of study (2 < fold changes < -2; a *p* < 0.05). Enrichment analysis for biological processes and immune system-related pathways and processes was performed using the PANTHER classification system and one quick analysis toll using the MetaCore software (Clarivate Analytics, Boston, MA, USA). For the immune system biological processes found in the MetaCore database (Clarivate Analytics), only the processes in which DEGs were grouped with a false discovery rate (FDR) < 0.01 were selected for analysis. DEGs associated with macrophage-related processes and functions were selected with the Database for Annotation Visualization and Integrated Discovery (DAVID) analysis.

### Proteomic shotgun analysis

PS and IpL samples were removed and rapidly frozen in liquid nitrogen. Total protein was extracted from D12 to 3dpp interpubic tissues using the PlusOne Sample Grinding Kit (GE Healthcare, Little Chalfont, Bucks, UK) in extraction buffer (4 M urea, 1 M thiourea, and 70 mM DTT). Buffer exchange into 50 mM ammonium bicarbonate was carried out using spin columns (Millipore, USA) and then quantified using a NanoDrop spectrophotometer. Samples were redigested with trypsin (Promega, USA) at a ratio of 1:50 (w/w trypsin/protein). The samples were suspended in water, and peptides were quantified using a Qubit Fluorometric Quantitation System (Thermo Fisher Scientific). Samples were diluted to 1 µg µL^−1^ and injected on a 2D ACQUITY UPLC M-Class system (Waters Corporation, Manchester, UK) coupled to a SYNAPT G2-Si Mass Spectrometer (Waters Corp.). Three micrograms of the sample were injected onto an XBridge BEH130 C18: 5 µm × 300 µm × 50 mm column (Waters Corp.) for peptide fractioning and elution to the analytical column. Peptides were eluted from the first column using three discontinued fractions containing 13.7%, 18.4%, and 50.0% acetonitrile on ammonium formate, pH = 10.

After fractionation and elution on the first column, peptides were separated on an HSS T3: 1.8 µm × 75 µm × 150 mm column (Waters Corp.) using a gradient of 7% to 40% acetonitrile/water containing 0.1% formic acid for 54 min, followed by 10 min of 85% acetonitrile/water at a flow rate of 0.4 µL min^−1^. Mass spectrometry was performed using nanoESI ionization in positive mode and data acquisition using HDMSE (Waters Corp.). The mass spectra were acquired from 50 to 2000 m/z. Protein identification and quantitation were performed using Progenesis QI for Proteomics v3.0 software (Waters Corp.).

The protein search was performed against the mouse revised databank from UniProt, using the following search parameters detailed by Silva and coworkers^73^. After identification, DEPs were associated with biological processes and immune system-related pathways and processes using the PANTHER classification system and one quick analysis toll in the MetaCore database (Clarivate Analytics). For immune system biological processes produced by MetaCore (Clarivate Analytics), only processes in which DEPs grouped with false discovery rate (FDR) < 0.01 were selected for analysis. DEPs associated with macrophage-related processes and functions or with immune system processes were selected for DAVID analysis.

### Real-time PCR (qPCR)

For gene expression analysis of complement system components *C2*, *C3*, *C5*, *C7,* and *Masp1*, total RNA was extracted from frozen D12 to 3dpp interpubic tissues using the RNeasy Kit (QIAGEN). After cDNA synthesis using a RevertAid H Minus First Strand cDNA Synthesis Kit (Fermentas, Maryland, USA) according to the manufacturer's recommendations, real-time PCR was performed using SYBR Green qPCR Master Mix (Applied Biosystems, Foster City, CA, USA) on an Applied Biosystems 7,300 Real-Time PCR System (Applied Biosystems). Each gene was normalized to the expression of the housekeeping gene 36b4. To quantify and acquire the fold increase in gene expression, the mathematical model 2-ΔΔCt was utilized as performed by Consonni and coworkers^9^, and the results were normalized to that of the D12. All primers (see Supplementary Table S6 online) were purchased from Applied Biosystems. Three (D18, D19, and 1dpp) or six animals (D12 and 3dpp) were used to obtain 200 ng of total RNA from each day of study, and all reactions were performed in triplicate on the same plate.

### Statistical analysis

The relative gene expression of qPCR analysis was performed based on Conover and Iman^74^ and Montgomery^75^ using rank data classification and semiparametric analysis (one-way ANOVA followed by Tukey's test or two-way ANOVA followed by Bonferroni's test) with *p* < 0.05 of significance performed by GraphPad Prism 5.0 (GraphPad Software, Inc., California, USA). All data are presented in graphs as the mean values ± standard error (SE).

## Supplementary information

Supplementary information

## References

[1] Sherwood, O. Relaxin. In *The Physiology of Reproduction.* (ed Neill E. Ka. J. D.) 861–1009 (Raven Press, New York, 1994).

[2] Mahendroo M (2012). Cervical remodeling in term and preterm birth: Insights from an animal model. Reproduction.

[3] Akgul Y, Holt R, Mummert M, Word A, Mahendroo M (2012). Dynamic changes in cervical glycosaminoglycan composition during normal pregnancy and preterm birth. Endocrinology.

[4] Ruscheinsky M, De la Motte C, Mahendroo M (2008). Hyaluronan and its binding proteins during cervical ripening and parturition: Dynamic changes in size, distribution and temporal sequence. Matrix Biol..

[5] Wieslander CK (2008). Regulation of elastolytic proteases in the mouse vagina during pregnancy, parturition, and puerperium. Biol. Reprod..

[6] Storey E (1957). Relaxation in the pubic symphysis of the mouse during pregnancy and after relaxin administration, with special reference to the behavior of collagen. J. Pathol. Bacteriol..

[7] Joazeiro, P. P., Consonni, S. R., Rosa, R. G. & Toledo, O. M. S. Peri-partum changes to mouse pubic symphysis. In *The Guide to Investigation of Mouse Pregnancy* 1st edn (eds Croy, A., Yamada, A. T., DeMayo, F. J. & Adamson, S. L.) 403–417 (Elsevier, Amsterdam, 2014).

[8] Rosa RG, Akgul Y, Joazeiro PP, Mahendroo M (2012). Changes of large molecular weight hyaluronan and versican in the mouse pubic symphysis through pregnancy. Biol. Reprod..

[9] Consonni SR (2012). Elastic fiber assembly in the adult mouse pubic symphysis during pregnancy and postpartum. Biol. Reprod..

[10] Pinheiro MC (2004). Histochemical and ultrastructural study of collagen fibers in mouse pubic symphysis during late pregnancy. Micron.

[11] Pinheiro MC (2005). Ultrastructural, immunohistochemical and biochemical analysis of glycosaminoglycans and proteoglycans in the mouse pubic symphysis during pregnancy. Cell Biol. Int..

[12] Consonni SR (2012). Recovery of the pubic symphysis on primiparous young and multiparous senescent mice at postpartum. Histol. Histopathol..

[13] Castelucci BG (2018). Time-dependent regulation of morphological changes and cartilage differentiation markers in the mouse pubic symphysis during pregnancy and postpartum recovery. PLoS ONE.

[14] Hall K (1954). Changes in the bone and cartilage of the symphysis pubis of the mouse during pregnancy and after parturition, as revealed by metachromatic staining and the periodic acid-schiff technique. J. Endocrinol..

[15] Borazjani A, Couri B, Balog B, Damaser M (2014). Mp1-13 pubic symphysis length is correlated with pelvic organ prolapse in lysyl oxidase like-1 knockout mice. J. Urol..

[16] Rosa RG (2011). Temporal changes in matrix metalloproteinases, their inhibitors, and cathepsins in mouse pubic symphysis during pregnancy and postpartum. Reprod. Sci..

[17] Garcia EA (2008). Hyaluronan involvement in the changes of mouse interpubic tissue during late pregnancy and postpartum. Cell Biol. Int..

[18] Gomez-Lopez N, StLouis D, Lehr MA, Sanchez-Rodriguez EN, Arenas-Hernandez M (2014). Immune cells in term and preterm labor. Cell Mol. Immunol..

[19] Timmons BC, Fairhurst AM, Mahendroo MS (2009). Temporal changes in myeloid cells in the cervix during pregnancy and parturition. J. Immunol..

[20] Rodriguez HA, Ortega HH, Ramos JG, Munoz-de-Toro M, Luque EH (2003). Guinea-pig interpubic joint (symphysis pubica) relaxation at parturition: Underlying cellular processes that resemble an inflammatory response. Reprod. Biol. Endocrinol..

[21] Shynlova O (2013). Infiltration of myeloid cells into decidua is a critical early event in the labour cascade and post-partum uterine remodelling. J. Cell Mol. Med..

[22] Timmons BC, Mahendroo MS (2006). Timing of neutrophil activation and expression of proinflammatory markers do not support a role for neutrophils in cervical ripening in the mouse. Biol. Reprod..

[23] Brown MB, von Chamier M, Allam AB, Reyes L (2014). M1/M2 macrophage polarity in normal and complicated pregnancy. Front. Immunol..

[24] Egashira M (2017). F4/80^+^ macrophages contribute to clearance of senescent cells in the mouse postpartum uterus. Endocrinology.

[25] Yellon SM (2017). Contributions to the dynamics of cervix remodeling prior to term and preterm birth. Biol. Reprod..

[26] Payne KJ, Clyde LA, Weldon AJ, Milford TA, Yellon SM (2012). Residency and activation of myeloid cells during remodeling of the prepartum murine cervix. Biol. Reprod..

[27] Zhang YH, He M, Wang Y, Liao AH (2017). Modulators of the balance between M1 and M2 macrophages during pregnancy. Front. Immunol..

[28] Zhang L (2011). The inflammatory changes of adipose tissue in late pregnant mice. J. Mol. Endocrinol..

[29] Mackler AM, Iezza G, Akin MR, McMillan P, Yellon SM (1999). Macrophage trafficking in the uterus and cervix precedes parturition in the mouse. Biol. Reprod..

[30] Nadeau-Vallee M (2016). Sterile inflammation and pregnancy complications: A review. Reproduction.

[31] Schaefer L (2014). Complexity of danger: The diverse nature of damage-associated molecular patterns. J. Biol. Chem..

[32] Frey H, Schroeder N, Manon-Jensen T, Iozzo RV, Schaefer L (2013). Biological interplay between proteoglycans and their innate immune receptors in inflammation. FEBS J..

[33] Wight TN (2020). Versican—A critical extracellular matrix regulator of immunity and inflammation. Front. Immunol..

[34] Couri BM (2017). Effect of pregnancy and delivery on cytokine expression in a mouse model of pelvic organ prolapse. Female Pelvic Med. Reconstr. Surg..

[35] Rosa RG (2008). Relaxation of the mouse pubic symphysis during late pregnancy is not accompanied by the influx of granulocytes. Microsc. Res. Tech..

[36] Castelucci BG, Consonni SR, Rosa VS, Joazeiro PP (2019). Recruitment of monocytes and mature macrophages in mouse pubic symphysis relaxation during pregnancy and postpartum recovery. Biol. Reprod..

[37] Zhao L (1999). Mice without a functional relaxin gene are unable to deliver milk to their pups. Endocrinology.

[38] Krajnc-Franken MA (2004). Impaired nipple development and parturition in LGR7 knockout mice. Mol. Cell Biol..

[39] Kaftanovskaya EM, Huang Z, Lopez C, Conrad K, Agoulnik AI (2015). Conditional deletion of the relaxin receptor gene in cells of smooth muscle lineage affects lower reproductive tract in pregnant mice. Biol. Reprod..

[40] Mi Y (2016). Functional consequences of mannose and asialoglycoprotein receptor ablation. J. Biol. Chem..

[41] Mizejewski GJ (2015). The alpha-fetoprotein third domain receptor binding fragment: In search of scavenger and associated receptor targets. J. Drug Target.

[42] Linck G, Petrovic A, Stoeckel ME, Porte A (1976). Fine structure of the public symphysis in the mouse. Bull. Assoc. Anat. (Nancy).

[43] Ricklin D, Hajishengallis G, Yang K, Lambris JD (2010). Complement: A key system for immune surveillance and homeostasis. Nat. Immunol..

[44] Chang MY (2017). Versican is produced by Trif- and type I interferon-dependent signaling in macrophages and contributes to fine control of innate immunity in lungs. Am. J. Physiol. Lung Cell Mol. Physiol..

[45] Barnett FH (2016). Macrophages form functional vascular mimicry channels in vivo. Sci. Rep..

[46] Passi A, Negrini D, Albertini R, Miserocchi G, De Luca G (1999). The sensitivity of versican from rabbit lung to gelatinase A (MMP-2) and B (MMP-9) and its involvement in the development of hydraulic lung edema. FEBS Lett..

[47] Gordon S, Taylor PR (2005). Monocyte and macrophage heterogeneity. Nat. Rev. Immunol..

[48] Moro CF, Consonni SR, Rosa RG, Nascimento MA, Joazeiro PP (2012). High iNOS mRNA and protein localization during late pregnancy suggest a role for nitric oxide in mouse pubic symphysis relaxation. Mol. Reprod. Dev..

[49] Chang MY (2012). Monocyte-to-macrophage differentiation: Synthesis and secretion of a complex extracellular matrix. J. Biol. Chem..

[50] Rayahin JE, Buhrman JS, Zhang Y, Koh TJ, Gemeinhart RA (2015). High and low molecular weight hyaluronic acid differentially influence macrophage activation. ACS Biomater. Sci. Eng..

[51] Schwabe J, Donnelly SM, Jesmin S, Leppert P, Mowa CN (2014). A proteomic profile of cervical remodeling in mice during early and late pregnancy. J. Steroids Horm. Sci..

[52] Stanley RL, Ohashi T, Gordon J, Mowa CN (2018). A proteomic profile of postpartum cervical repair in mice. J. Mol. Endocrinol..

[53] Lutz PG, Houzel-Charavel A, Moog-Lutz C, Cayre YE (2001). Myeloblastin is an Myb target gene: Mechanisms of regulation in myeloid leukemia cells growth-arrested by retinoic acid. Blood.

[54] Klimiankou M, Mellor-Heineke S, Zeidler C, Welte K, Skokowa J (2016). Role of CSF3R mutations in the pathomechanism of congenital neutropenia and secondary acute myeloid leukemia. Ann. N. Y. Acad. Sci..

[55] Sharma P, Sharma A, Srivastava M (2017). In vivo neutralization of alpha4 and beta7 integrins inhibits eosinophil trafficking and prevents lung injury during tropical pulmonary eosinophilia in mice. Eur. J. Immunol..

[56] Ortega-Gomez A (2016). Cathepsin G controls arterial but not venular myeloid cell recruitment. Circulation.

[57] Janssens R, Struyf S, Proost P (2018). The unique structural and functional features of CXCL12. Cell Mol. Immunol..

[58] Girardi G (2018). Complement activation, a threat to pregnancy. Semin. Immunopathol..

[59] Mastellos DC, Deangelis RA, Lambris JD (2013). Complement-triggered pathways orchestrate regenerative responses throughout phylogenesis. Semin. Immunol..

[60] Luo C, Chen M, Madden A, Xu H (2012). Expression of complement components and regulators by different subtypes of bone marrow-derived macrophages. Inflammation.

[61] Modinger Y, Loffler B, Huber-Lang M, Ignatius A (2018). Complement involvement in bone homeostasis and bone disorders. Semin. Immunol..

[62] Stubelius A (2016). Ncf1 affects osteoclast formation but is not critical for postmenopausal bone loss. BMC Musculoskelet. Disord..

[63] Kang JH, Sim JS, Zheng T, Yim M (2017). F4/80 inhibits osteoclast differentiation via downregulation of nuclear factor of activated T cells, cytoplasmic 1%. J. Arch. Pharm. Res..

[64] Merle NS, Noe R, Halbwachs-Mecarelli L, Fremeaux-Bacchi V, Roumenina LT (2015). Complement system part II: Role in immunity. Front. Immunol..

[65] Veridiano AM (2007). The mouse pubic symphysis as a remodeling system: Morphometrical analysis of proliferation and cell death during pregnancy, partus and postpartum. Cell Tissue Res..

[66] Hong Q, Kuo E, Schultz L, Boackle RJ, Chang NS (2007). Conformationally altered hyaluronan restricts complement classical pathway activation by binding to C1q, C1r, C1s, C2, C5 and C9, and suppresses WOX1 expression in prostate DU145 cells. Int. J. Mol. Med..

[67] Groeneveld TW (2005). Proteoglycans decorin and biglycan with C1q interactions of the extracellular matrix and collectins. J. Immunol..

[68] Fingleton B (1864). Matrix metalloproteinases as regulators of inflammatory processes. Biochim. Biophys. Acta Mol. Cell Res..

[69] Bellac CL (2014). Macrophage matrix metalloproteinase-12 dampens inflammation and neutrophil influx in arthritis. Cell Rep..

[70] Bennett HS, Wyrick AD, Lee SW, McNeil JH (1976). Science and art in preparing tissues embedded in plastic for light microscopy, with special reference to glycol methacrylate, glass knives and simple stains. Stain Technol..

[71] Joazeiro, P. P., Consonni, S. R., Rosa, R. G. & Toledo, O. M. S. Pubic symphysis evaluation. In *The Guide to Investigation of Mouse Pregnancy* 1st edn (eds Croy, A., Yamada, A. T., DeMayo, F. J. & Adamson, S. L.) 733–749 (Elsevier, Amsterdam, 2014).

[72] Bruni-Cardoso A, Vilamaior PS, Taboga SR, Carvalho HF (2008). Localized matrix metalloproteinase (MMP)-2 and MMP-9 activity in the rat ventral prostate during the first week of postnatal development. Histochem. Cell Biol..

[73] Silva JC (2005). Quantitative proteomic analysis by accurate mass retention time pairs. Anal. Chem..

[74] Conover WJ, Iman RL (1981). Rank transformations as a bridge between parametric and nonparametric statistics. Am. Stat..

[75] Montgomery DC (1991). Design and Analysis of Experiments.

