# Supplementary material for: Evidence of macrophage modulation in the mouse pubic symphysis remodeling during the end of first pregnancy and postpartum
Source: Sci Rep. 2020 Jul 24;10:12403. doi: 10.1038/s41598-020-68676-x (PMC7381608; doi:10.1038/s41598-020-68676-x)
Supplement: Supplementary file 1 — Supplementary information [file 41598_2020_68676_MOESM1_ESM.pdf]

Title: Evidence of macrophage modulation in the mouse pubic symphysis remodeling during the end of first pregnancy and postpartum

Authors: BG Castelucci[1], AHM Pereira[2], M Fioramonte[,3], MF Carazzolle[4], PSL de Oliveira[2], KG Franchini[2], J Kobarg[5], D Martins-de-Souza[3,6,7], PP Joazeiro[1], SR Consonni[1*].

Supplementary Table S1. List of biological processes related to immune system response in with DEGs and DEPs (-2 > fold changes > 2) were grouped by MetaCore enrichment analysis, detailing their FDR and *p-value* per day of study (n=3/group).

	**Biological processes related to immune system response**	**D18**		**D19**		**1dpp**		**3dpp**	
		p-value	*FDR*	*p-value*	*FDR*	*p-value*	*FDR*	*p-value*	*FDR*
**DEGs**	Classical complement pathway	6.78E-01	8.35E-01	4.95E-01	9.20E-01	1.47E-05	1.40E-03	2.35E-07	1.95E-05
	Lectin induced complement pathway	2.80E-01	8.35E-01	2.01E-01	9.20E-01	3.65E-05	1.73E-03	9.32E-07	3.87E-05
	Transcription regulation of granulocyte development	4.94E-01	8.35E-01	6.38E-01	9.20E-01	5.51E-05	1.74E-03	2.44E-03	5.16E-02
	Role of proteases in hematopoietic stem cell mobilization	5.26E-02	7.00E-01	1.05E-01	9.20E-01	1.64E-04	3.90E-03	7.17E-03	9.80E-02
**DEPs**	Phagocytosis	2.16E-01	4.38E-01	6.01E-02	2.46E-01	3.89E-03	5.25E-02	5.95E-03	8.41E-02

Supplementary Table S2. List of DEGs (-2 > fold changes > 2) associated with transcriptional regulation of granulocyte development by MetaCore enrichment analysis for biological processes, detailing their molecular function and fold changes from D18 to 3dpp (n=3/group; FDR < 10^{-3}; p < 0.0001).

Ensembl number	Gene ID	Gene name	Molecular function	Fold change			
				D18	D19	1dpp	3dpp
ENSMUST00000049004	*Anpep*	Alanyl (membrane) aminopeptidase	Metalloprotease	-	-	2.84	2.71
ENSMUST00000030673	*Csf3r*	Colony stimulating factor 3 receptor (granulocyte)	Generic receptor	-	-	-2.29	-
ENSMUST00000015484	*Cybb*	Cytochrome b-245. beta polypeptide	Generic enzyme	-	-	-2.55	-
ENSMUST00000046091	*Elane*	Elastase. neutrophil expressed	Generic protease	-	-	-7.52	-2.30
ENSMUST00000033502	*Gata1*	GATA binding protein 1	Transcription factor	-	-		-2.11
ENSMUST00000035077	*Ltf*	Lactotransferrin	Transporter	-	-	-6.56	-
ENSMUST00000020779	*Mpo*	Myeloperoxidase	Generic enzyme	-	-	-7.99	-2.40
ENSMUST00000001184	*Mxd1*	MAX dimerization protein 1	Transcription factor	-	-	-2.57	-
ENSMUST00000020158	*Myb*	Myeloblastosis oncogene	Transcription factor	-2.20	-2.10	-6.97	-3.63
ENSMUST00000016094	*Ncf1*	Neutrophil cytosolic factor 1	Generic binding protein	-	-	-2.12	-
ENSMUST00000006679	*Prtn3*	Proteinase 3	Generic protease	-	-	-3.33	-2.21

Supplementary Table S3. List of DEGs (-2 > fold changes > 2) associated with the role of proteases in hematopoietic stem cell mobilization by MetaCore enrichment analysis for biological processes, detailing their molecular function and fold changes from D18 to 3dpp (n=3/group; FDR < 10^{-3}; $p < 0.0001$).

**Ensembl number**	**Gene ID**	**Gene name**	**Molecular function**	**Fold change**			
				D18	D19	1dpp	3dpp
ENSMUST00000015583	*Ctsg*	Cathepsin G	Generic protease	-	-	-4.06	-2.31
ENSMUST00000073043	*Cxcl12*	Chemokine (C-X-C motif) ligand 12	Receptor ligand	-	-	-2.56	-
ENSMUST00000052172	*Cxcr4*	Chemokine (C-X-C motif) receptor 4	GPCR	-	-	-3.39	-
ENSMUST00000046091	*Elane*	Elastase. neutrophil expressed	Generic protease	-	-	-7.52	-2.30
ENSMUST00000055226	*Fn1*	Fibronectin 1	Receptor ligand	2.68	2.41	2.50	2.39
ENSMUST00000099972	*Itga4*	Integrin alpha 4	Generic receptor	-	-2.08	-2.77	-2.35
ENSMUST00000034187	*Mmp2*	Matrix metallopeptidase 2	Metalloprotease	3.02	2.75	2.70	2.73
ENSMUST00000017881	*Mmp9*	Matrix metallopeptidase 9	Metalloprotease	-	-	-2.40	-

Supplementary Table S4. List of primary antibodies used for double immunostaining assays.

Primary antibodies	Description	Dilution
Anti-BM8 (T-2028.0100)	Rat monoclonal antibody from BACEM Biosciences Inc. (King of Prussia, PA).	1:300
Anti-Decorin (ab137508)	Rabbit polyclonal antibody from Abcam plc. (Cambridge, MA, USA).	1:300
Anti-Versican (sc25831)	Rabbit polyclonal antibody from Santa Cruz Biotechnology Inc. (California, USA).	1:300
Anti-Hyaluronic acid (ab53842)	Sheep polyclonal antibody from Abcam plc. (Cambridge, MA, USA).	1:300
Anti-VEGFR2	Rabbit polyclonal antibody from eBioscience (San Diego, CA, USA).	1:300

Supplementary Table S5. List of secondary antibodies used for double immunostaining assays.

Secondary antibodies	Description	Dilution
Alexa Fluor 647 (ab150155)	Donkey antibody anti-rat IgG from Abcam plc. (Cambridge, MA, USA).	1:500
Alexa Fluor 488 (ab150179)	Donkey antibody anti-rat IgG from Abcam plc. (Cambridge, MA, USA).	1:500
Alexa Fluor 647 (ab150179)	Donkey antibody anti-sheep IgG from Abcam plc. (Cambridge, MA, USA).	1:500
Alexa Fluor 488 (ab150077)	Goat antibody anti-rabbit IgG from Abcam plc. (Cambridge, MA, USA).	1:500

Supplementary Table S6. List of primers used in the qPCR analysis.

Gene	Primer sequence	Amplicon	NCBI reference sequence
36b4	F: CACTGGTCTAGGACCCGAGAAG<br>R: GGTGCCTCTGGAGATTTTCG	72	NM_007475.5
C2	F: CTCATCCGCGTTTACTCCAT<br>R: TGTTCTGTTCGATGCTCAGG	178	NM_013484.2
C3	F: AGCAGGTCATCAAGTCAGGC<br>R: GATGTAGCTGGTGTTGGGCT	167	NM_009778.3
C5	F: AGGGTACTTTGCCTGCTGAA<br>R: TGTGAAGGTGCTCTTGGATG	173	NM_010406.2
C7	F: CCTTGGATGTATGTGCCCGA<br>R: ACCACAGACTTTCTCAGCCG	152	NM_001243837.1
Masp1	F: AGGACCTGCCGAGTGGAATG<br>R: TCTCCACAGAAGGGACCCCA	251	NM_001359083.1
